# A Case Study of Genomic Instability in an Industrial Strain of *Saccharomyces cerevisiae*

**DOI:** 10.1534/g3.118.200446

**Published:** 2018-09-25

**Authors:** Aline Rodrigues-Prause, Nadia M. V. Sampaio, Theodore M. Gurol, Guadalupe M. Aguirre, Hailey N. C. Sedam, Mary J. Chapman, Ewa P. Malc, V. P. Ajith, Parijat Chakraborty, Pedro A. Tizei, Gonçalo A. G. Pereira, Piotr A. Mieczkowski, Koodali T. Nishant, Juan Lucas Argueso

**Affiliations:** * Department of Environmental and Radiological Health Sciences; ‡ Cell and Molecular Biology Graduate Program, Colorado State University, Fort Collins-CO 80523; † Departamento de Genética, Evolução e Bioagentes, Instituto de Biologia, Universidade Estadual de Campinas, Campinas-SP, Brazil; § Department of Genetics, University of North Carolina, Chapel Hill-NC 27599; ** School of Biology, Indian Institute of Science Education and Research; †† Center for Computation Modeling and Simulation, Indian Institute of Science Education and Research, Thiruvananthapuram, Trivandrum 695016, India

**Keywords:** *Saccharomyces cerevisiae*, Loss-of-heterozygosity, *ACE2*, Fermentation, Colony morphology, Mitotic recombination

## Abstract

The *Saccharomyces cerevisiae* strain JAY270/PE2 is a highly efficient biocatalyst used in the production of bioethanol from sugarcane feedstock. This strain is heterothallic and diploid, and its genome is characterized by abundant structural and nucleotide polymorphisms between homologous chromosomes. One of the reasons it is favored by many distilleries is that its cells do not normally aggregate, a trait that facilitates cell recycling during batch-fed fermentations. However, long-term propagation makes the yeast population vulnerable to the effects of genomic instability, which may trigger the appearance of undesirable phenotypes such as cellular aggregation. In pure cultures of JAY270, we identified the recurrent appearance of mutants displaying a mother-daughter cell separation defect resulting in rough colonies in agar media and fast sedimentation in liquid culture. We investigated the genetic basis of the colony morphology phenotype and found that JAY270 is heterozygous for a frameshift mutation in the *ACE2* gene (*ACE2/ace2-A7*), which encodes a transcriptional regulator of mother-daughter cell separation. All spontaneous rough colony JAY270-derived isolates analyzed carried copy-neutral loss-of-heterozygosity (LOH) at the region of chromosome XII where *ACE2* is located (*ace2-A7/ace2-A7*). We specifically measured LOH rates at the *ACE2* locus, and at three additional chromosomal regions in JAY270 and in a conventional homozygous diploid laboratory strain. This direct comparison showed that LOH rates at all sites were quite similar between the two strain backgrounds. In this case study of genomic instability in an industrial strain, we showed that the JAY270 genome is dynamic and that structural changes to its chromosomes can lead to new phenotypes. However, our analysis also indicated that the inherent level of genomic instability in this industrial strain is normal relative to a laboratory strain. Our work provides an important frame of reference to contextualize the interpretation of instability processes observed in the complex genomes of industrial yeast strains.

Over the last decade, the genomic characterization of many diverged diploid isolates of the yeast *Saccharomyces cerevisiae* has revealed the existence of substantial intra-strain variability in nucleotide sequence and chromosome structure (Argueso *et al.* 2009; Borneman *et al.* 2011; Magwene *et al.* 2011; Peter *et al.* 2018). While most conventional laboratory strains, when diploidized, harbor nearly completely homozygous genomes and display stable phenotypes, the genome architecture of wild strains, including clinical and industrial isolates, has often been found to be more complex, with abundant inter-homolog nucleotide heterozygosity and structural variation.

In several species, widespread heterozygosity is often associated with beneficial phenotypes. It is suspected that this genomic configuration may also contribute to the adaptation of wild yeasts to their respective habitats, as well as to the robust growth, stress tolerance, and other desirable traits characteristic of industrial yeasts (Muller and McCusker 2009; Plech *et al.* 2014). Interestingly, the maintenance of heterozygosity by these single-celled organisms is challenging because allelic mitotic recombination events between homologous chromosomes occurs at relatively high rates (∼10^−4^/cell division; (St Charles and Petes 2013)), and can lead to immediate fixation of tracts of loss-of-heterozygosity (LOH) in the clonal cell lineage. Accordingly, in JAY270/PE2 and other wild strains, heterozygous regions are interspersed with stretches of homozygosity indicating the occurrence of LOH events in clonal ancestors (Magwene *et al.* 2011). In addition, multiple studies have characterized the emergence of chromosomal rearrangements in cells clonally derived from industrial strains (Nadal *et al.* 1999; Carro and Pina 2001; Basso *et al.* 2008; Querol and Bond 2009; de Amorim *et al.* 2017). Those detailed studies, combined with informal field observations occasionally reported by operators of wine, beer and biofuel fermentations, have earned industrial yeast strains a reputation for having unstable genomes. This genomic instability could, and likely does, make fermentations that use such strains vulnerable to process disruption due to the loss of selected traits or appearance of undesirable phenotypes.

One of the first heterozygous *S. cerevisiae* strains to have its genome characterized was JAY270/PE2, widely used in sugarcane bioethanol production (Argueso *et al.* 2009). This heterothallic diploid was originally isolated at a Brazilian distillery as an aggressive, yet highly productive wild contaminant (Basso *et al.* 2008). One of its most attractive features is its suitability to continuous cell recycling in batch-fed fermentations. JAY270/PE2 offers distillery operators predictable fermentation conditions during the 8-9 month sugarcane harvest season, because it is often able to outcompete wild yeast contaminants that are steadily introduced through the feedstock into the recycling system.

 Here, we report a case study of two easily discernible and related mutant phenotypes that result from a specific rearrangement of the JAY270/PE2 genome. We characterized the spontaneous appearance of clones displaying a mother-daughter cell separation defect among pure cultures of this strain. This cell separation defect resulted in both rough colony morphology in agar plates and fast sedimentation in liquid culture. We found that these phenotypes were recurrently caused by an LOH event spanning the *ACE2* locus. We measured the quantitative rate and characterized the qualitative spectrum of mitotic recombination at the region of chromosome XII (Chr12) where *ACE2* is located. In order to provide reference for a broader comparison of genomic instability levels, we also measured the rates of LOH at three additional independent chromosomes, using both JAY270/PE2 and a conventional diploid homozygous laboratory strain. We found that the rates of mitotic recombination were quite similar overall between the four genomic regions and between the two strain backgrounds. These results showed that, while chromosomal rearrangements did occur at detectable rates and could lead to new phenotypes in JAY270/PE2, its inherent level of genomic instability was not elevated relative to the reference strain. Instead, we found that the genome of the conventional laboratory strain was just as dynamic as to that of JAY270/PE2.

## Materials and Methods

### Yeast genetic backgrounds and culture procedures

*Saccharomyces cerevisiae* strains used in this study descended from either the JAY270 or CG379 strain backgrounds (Table S1). JAY270 is a heterothallic diploid single colony isolate derived from the industrial bioethanol strain PE2 (Argueso *et al.* 2009). CG379 diploid is related to the S288c laboratory strain background (Morrison *et al.* 1991; Argueso *et al.* 2008). 

Yeast cells were grown in YPD (20 g/L glucose, 20 g/L peptone, 10 g/L yeast extract, 20 g/L bacteriological agar for solid media) and synthetic media (20 g/L glucose, 5 g/L ammonium sulfate, 1.7 g/L yeast nitrogen base without amino acids, 1.4 g/L complete drop-out mix, 20 g/L bacteriological agar). Standard procedures for yeast transformation, crossing and sporulation were followed (Ausubel *et al.* 1998). Counter-selections against *URA3* were performed in synthetic media supplemented with 1g/L of 5-Fluoroorotic Acid (5-FOA).

### Allele replacement and complementation tests

A two-step allele replacement plasmid was constructed by cloning a segment of the wild type *ACE2* allele containing the 8-adenine homopolymer into the *URA3* pRS306 integrative vector (Sikorski and Hieter 1989). First, pRS306 was digested with *Bam*HI and *Kpn*I and ligated to a *Bgl*II- and *Kpn*I-digested segment of the wild type *ACE2* allele (PCR amplified from JAY289 genomic DNA using primers JAO904 and JAO905). The resulting plasmid, pAR1, was linearized with *Eco*RI and transformed into 5-FOA resistant (*ura3* mutant) derivatives of JAY291 and JAY292 haploids (both mutant *ace2-A7*). Pop-in Ura+ integration transformants were selected, and pop-out events were then selected for resistance to 5-FOA. 5-FOA resistant candidates were screened by PCR and Sanger sequencing to verify the presence of the wild type *ACE2* allele. This procedure resulted in the *ACE2* strains JAY1051 and JAY1039, isogenic to JAY291 and JAY292 respectively. Complementation tests using these strains were performed as shown in Figure 2C.

### Construction of strains used in LOH assays

The JAY270 strains used in the LOH assays were constructed from a homozygous *ura3Δ/ura3Δ* derivative of JAY270 (JAY585; gift from F. Galzerani). The CORE2 cassette containing the *Kluveromyces lactis URA3* gene, the *S. cerevisiae URA3* gene and the *Kan*MX4 geneticin resistance marker (*_Kl_URA3-_Sc_URA3-KanMX4*) was amplified from pJA40 (Zhang *et al.* 2013) with primers targeting integration at four different genomic regions (Table S2). Integration in the maternal or paternal Chr12 homolog was checked by PCR and Sanger genotyping of linked HetSNPs. The same procedures and primers were used integrate CORE2 in the CG379 background. 

### Quantitative LOH rate assays

Diploid yeast cells hemizygous for CORE2 insertions were streaked to single colonies on solid YPD medium and incubated at 30° for two days. Single colonies were inoculated into 5 ml liquid YPD, and incubated for 24 hr at 30° in a rotating drum. The cultures were serially diluted and plated on YPD (permissive) or 5-FOA (selective) media. Colonies were counted after 2 days of growth on permissive and 4 days on selective plates, and colony counts were used to calculate recombination rates and 95% confidence intervals using the Lea & Coulson method of the median within the FALCOR web application (http://www.keshavsingh.org/protocols/FALCOR.html) (Lea and Coulson 1949; Hall *et al.* 2009). Statistical analyses of pairwise comparisons between recombination rates were performed using a two-sided nonparametric Mann Whitney test in GraphPad Prism software.

### Genotyping specific HetSNPs Through PCR-RFLP and molecular karyotyping

Genotyping was performed by PCR-amplification of regions containing the HetSNPs followed by restriction digestion and agarose gel analysis fragment length polymorphisms. In cases of markers where the HetSNPs were not associated with an RFLP, PCR products were Sanger-sequenced. A complete list of the HetSNPs coordinates, primers and restriction enzymes used is provided in Table S3. 

### Genome Sequencing Analyses

The genomes of the JAY270 parent strain and of 56 haploids derived from 14 complete JAY270 tetrads were sequenced using the Illumina short read whole genome sequencing platform. The sequencing reads were used to create a phased draft map of high confidence HetSNPs in JAY270’s genome (detailed information about the pipeline used are provided in the Supplemental Material). Genome sequencing data associated with this study is available in the Sequence Read Archive (SRA) database under study number SRP082524.

### Quantitative digital droplet PCR

 Genomic DNA was prepared from PFGE agarose plugs as previously described (Zhang *et al.* 2013). Sheared DNA was then digested with *Mfe*I-HF, a restriction enzyme that cuts the genome frequently, but does not cut within the ddPCR amplicons. Digested DNA was diluted to 0.05 ng/μl and 2 μl of this dilution was used as a template for each reaction using BioRad’s QX200 EvaGreen Supermix. The internal genomic DNA template normalizer was the average between signal from *DNM1* and *YLR001C* (regions of known two copy number), divided by 2.

### Data and reagent availability

Strains and plasmids are available upon request. Supplemental files available at FigShare. Sequence data are available at the Sequence Read Archive (SRA) database under study number SRP082524. Supplemental material available at Figshare: https://doi.org/10.25387/g3.6406598.

## Results

### Spontaneous JAY270 derivatives display a recurrent cell separation defect

**Figure 1:**
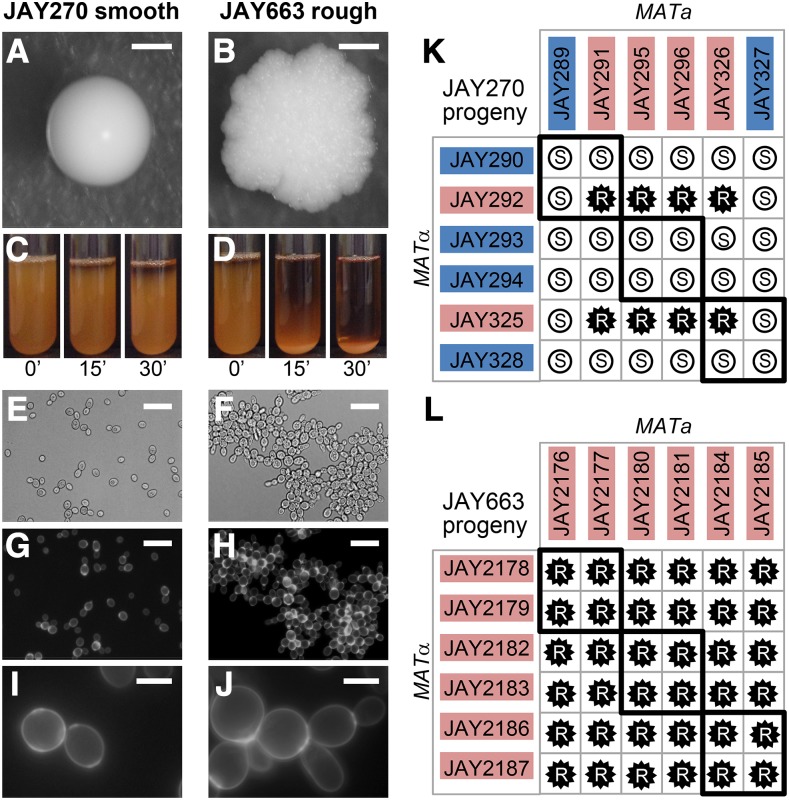
Smooth and rough colony morphologies, liquid sedimentation, mother-daughter cell attachment, and phenotypes of diploids derived from mating specific haploids. A-J show images of the JAY270 smooth parent diploid strain (left panels) and its spontaneous rough derivative JAY663 (right panels). A and B, colony morphologies on YPD agar after 3 days growth at 30C. C and D, cell sedimentation kinetics. 5 ml liquid YPD cultures were grown overnight at 30C in a rotating drum. Test tubes were vortexed vigorously for 10 sec to fully resuspend the cells, then were left to rest and photographed at 15 min intervals (the 0’ pictures were taken immediately after vortexing). E and F, bright field, and G-J, fluorescence microscopy of cells stained with calcofluor white to highlight chitin septa and the mother-daughter cell attachment. Scale bars are 1mm (A-B), 20µm (E-F) and 5µm (G-J). K and L, Smooth (S, white circles) and rough (R, black stars) phenotypes of diploids formed by crossing the indicated *MATa* and *MATα* haploids isolated from three tetrads of each JAY270 (K) and JAY663 (L). Thick black lines indicate the four diploids derived from matings of intra-tetrad sibling haploids. The colored backgrounds for each haploid correspond to their inferred genotype (Blue, dominant wild type allele; Red, recessive mutant allele). All 12 haploids from panel K had their whole genomes sequenced. Co-segregation analysis with JAY270 HetSNPs (Fig. S1) was used for identification of the causal mutation at the *ACE2* locus (Fig. 2).

Yeast cellular aggregation can be a problem in batch-fed sugarcane bioethanol production because it reduces fermentation kinetics and leads to clogging of the centrifuges and pipes used to recycle the yeast biomass from one tank to the next (Della-Bianca *et al.* 2013; Paulino de Souza *et al.* 2018). One of the desirable industrial traits of the JAY270/PE2 strain (hereafter referred to simply as JAY270) is that its cells do not usually aggregate (Basso *et al.* 2008). Accordingly, JAY270 produces normal hemispherical colonies with smooth surfaces and edges when grown in solid agar medium, and remains in dispersed suspension in liquid culture for prolonged periods (Figure 1A and 1C). While these are the phenotypes typically observed, over the course of our studies using this strain we noticed the sporadic occurrence of colonies clonally derived from JAY270 that displayed altered morphology: relatively flat-growing colonies with rough surfaces and edges (Figure 1B). In addition, when grown in liquid media, these clones sedimented at a faster rate compared to JAY270 (Figure 1D). Under bright field microscopic examination, yeast cells derived from such rough colonies appeared to grow in chains, showing a budding pattern consistent with a defect in the separation of the daughter cells from their mother (Figure 1E-F). We stained these cells with calcofluor white to visualize the chitin-rich ring septa, confirming the persistent attachment of mother and daughter cells at the budding neck site (Figure 1G-J).

We initially isolated five independent examples of such rough colonies for genetic characterization (JAY663, JAY664, JAY665, JAY912 and JAY913), all of which were derived either directly from JAY270 or from JAY270-isogenic strains. The rough colony phenotype of these isolates was stably maintained and was not reversible over several clonal generations, suggesting that it was likely encoded genetically and not caused by a transient transcriptional or post-transcriptional state. We estimated that these five isolates appeared spontaneously from a pool of ∼5 × 10^4^ smooth colonies. Assuming a genetic origin and based on this high frequency of occurrence in diploid cells, we reasoned that this phenotype was unlikely to be caused by a rare dominant *de novo* nucleotide point mutation, which might be expected to occur in the 10^−9^-10^−8^/cell division rate range. Instead, a more plausible mutational mechanism was mitotic recombination leading to loss-of-heterozygosity (LOH), because it occurs at a compatible rate (∼10^−4^/cell division; (St Charles and Petes 2013)) and would not require a dominant phenotypic effect.

### Investigation of the genetic basis of the rough colony phenotype

In a parallel project, we observed that crossing two specific haploid descendants of JAY270 (JAY291 *MATa* and JAY292 *MATα*; (Argueso *et al.* 2009)) resulted in diploid cells that displayed rough colony morphology and fast sedimentation phenotypes indistinguishable from those exhibited by the five isolates described above. Analysis of the JAY291 and JAY292 haploids showed that both displayed a mother-daughter cell separation defect and fast sedimentation, but neither had the rough colony phenotype (data not shown). JAY291, JAY292 and all other haploid derivatives of JAY270 display the normal smooth colony morphology.

The ability to consistently reproduce the mutant colony morphology phenotype in controlled crosses between specific haploids opened an avenue to investigate its genetic basis. We pursued this approach by carrying out crosses between twelve haploid descendants of JAY270 (3 tetrads; Figure 1K), all of which had their whole genomes sequenced (see below). All possible *MATa* x *MATα* crosses were performed producing 36 different diploids. Among them, we found eight with rough and 28 with smooth colony surfaces, in a pattern that was consistent with recessive inheritance of a trait controlled by a single gene. Even though the rough colony phenotype was not observed in any of the parents, the phenotypes of the respective diploids allowed us to infer which allele was present in the haploids: either the wild type dominant allele or the recessive mutant allele.

In addition, for comparison, we conducted a similar tetrad dissection and mating analysis with one of the spontaneous rough-colony isolates, JAY663. We examined the phenotypes of its haploid derivatives and found that none displayed the rough colony phenotype; they were all smooth (∼100 examined). We then took twelve of these haploids comprising three full tetrads (JAY2176 through JAY2187), determined their mating types, and conducted all possible mating combinations between them (Figure 1L). In this case, all 36 crosses resulted in rough colony diploids, indicating that all twelve haploid parents carried the causal recessive mutant allele, and therefore, JAY663 must have been homozygous for it. This was consistent with the hypothesis that copy-neutral LOH might be responsible for the sporadic appearance of the mutant morphology phenotype in JAY270 clonal derivatives.

### Rough colony phenotype results from loss of the wild type *ACE2* allele in diploids

**Figure 2:**
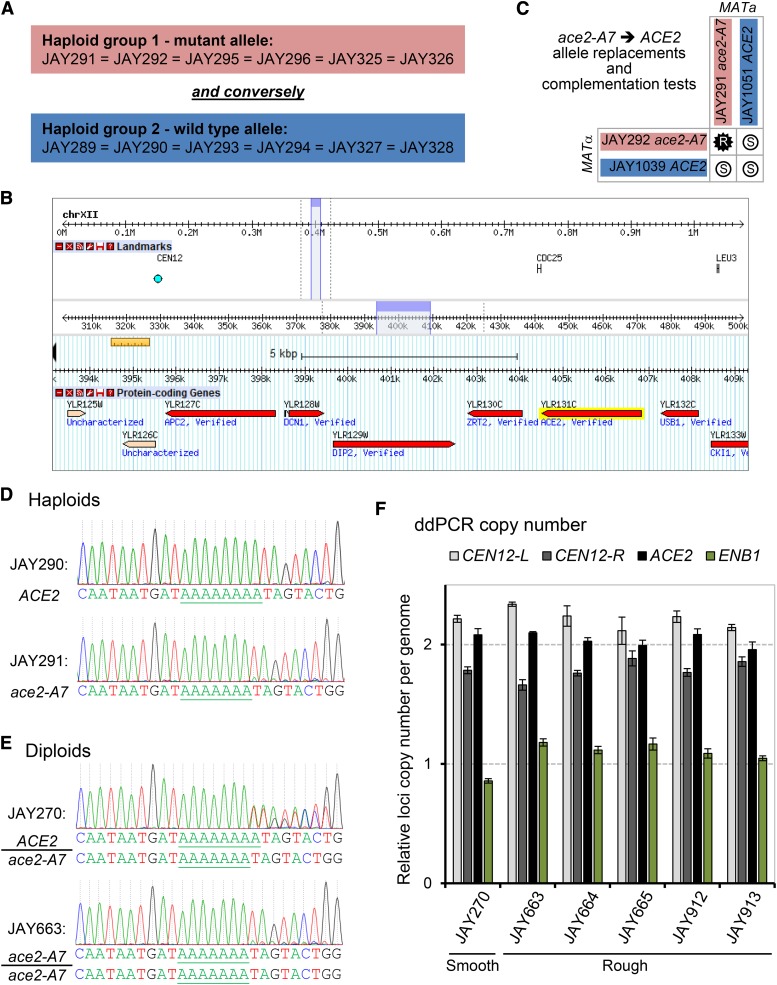
Mapping and characterization of the *ace2-A7* mutation. A The genetic mapping approach consisted of identifying HetSNP linkage regions in which one allele (M or P) co-segregated in all six haploids inferred to carry the rough mutant allele (red), while the other allele (P or M) co-segregated in all six haploids inferred to contain the smooth wild type allele (blue) (see Fig. 1K). B Genome Browser view of a Chr12 candidate region that satisfied the strict co-segregation criterion. This region spans 13 Kb and contains 9 genes (from *YLR125W* to *CKI1*). Review of the functional annotations of the genes in this region identified the *ACE2* locus (highlighted in yellow) as a likely candidate, and further sequence review uncovered the *ace2-A7* mutation. A second candidate region on Chr11 (not shown) was also identified through co-segregation analysis, but was not pursued further. C Allele replacements and complementation tests. The *ace2-A7* allele from two haploid strains JAY291 (*MATa)* and JAY292 (*MATα*) was replaced with the *ACE2* allele, generating JAY1051 and JAY1039, respectively. Matings between the *ace2-A7* and *ACE2* haploids confirmed the restoration of the smooth phenotype in diploid strains derived from at least one haploid parent containing the *ACE2* allele. D and E Sanger DNA sequencing analysis of *ACE2* locus. D shows segments of PCR-Sanger sequencing chromatograms for the *ACE2* locus from haploids with the wild type *ACE2* (JAY290) and mutant *ace2-A7* (JAY291) alleles. Note the difference in the A-homopolymer runs, with eight consecutive peaks in *ACE2* and seven consecutive peaks in *ace2-A7*. E shows PCR-Sanger chromatograms from diploids (primer extension was from left to right in all cases). The JAY270 chromatogram is a mixture of the two alleles (heterozygous), while the rough colony clone JAY663 only had the *ace2-A7* pattern. The inferred DNA sequences in D and E are shown below the chromatograms. The chromatograms from rough clones JAY664, JAY665, JAY912 and JAY913 were indistinguishable from JAY663 (not shown). F Gene copy number analysis of the *ACE2* locus. Plots show the calculated copy number for the four genomic regions probed, relative to the average of the signal between the control probes flanking the Chr12 centromere (*CEN12-L* and *CEN12-R*), both known to be present in two copies in the JAY270 genome. The *ENB1* region was another control probe, known to be present as one copy in JAY270.

The results described above suggested that the rough colony phenotype was diploid-specific, and caused by a recessive mutation present in heterozygous state in JAY270. Based on this premise, we divided the twelve sequenced JAY270-derived haploids from Figure 1K into two groups according to their inferred genotype (Figure 2A). Group 1 included the six haploids inferred to carry the mutant recessive allele of the causal gene, whereas group 2 included the six haploids with the wild type dominant allele.

We previously reported the whole genome sequence of the JAY291 haploid (Argueso *et al.* 2009). Since then we have sequenced the genomes of 55 additional JAY270-derived haploids, including the haploids used in the crosses shown in Figure 1K. This genome sequence dataset, comprising fourteen sets of four-spore tetrads, was generated in a project to characterize the abundance, distribution and phasing of heterozygous loci in the JAY270 genome, the full results of which will be described elsewhere. These haploid genomic sequences were used to create a draft map of phased heterozygous single nucleotide polymorphisms (HetSNPs) containing 12,023 loci unevenly distributed across the genome (Fig. S1).

In order to map the gene underlying the colony morphology trait, we searched for the JAY270 HetSNPs that co-segregated among the haploids from groups 1 or 2. We interrogated each of the genome-wide HetSNPs, and determined which had alleles that co-segregated in all six individuals within group 1, and conversely, had the other allele co-segregating in all six individuals within group 2. This analysis identified only two candidate regions that fit the strict co-segregation criterion. One of the candidate regions corresponded to ∼30 Kb on Chr11, including thirteen genes (not shown); and the other spanned ∼15 Kb containing nine genes on the right arm of Chr12 (Figure 2B), located ∼50 Kb centromere proximal to the ribosomal DNA tandem repeats (rDNA). 

We reviewed the annotations of the 22 candidate genes, and identified a gene located in the Chr12 region, *ACE2*, which encodes a transcription factor that controls the expression of genes involved in the mother-daughter cell separation process (Weiss 2012). In cells lacking Ace2p, the daughter cell remains attached to the mother cell at the bud neck, resulting in the accumulation of multicellular clusters. These clusters lead to faster sedimentation in liquid culture compared to dispersed cells. Importantly, while both haploid *ace2* and diploid *ace2*/*ace2* mutants form multicellular clusters and display faster sedimentation in liquid culture, only the diploids form rough colonies in agar media (Voth *et al.* 2005). Haploid *ace2* mutants form normal smooth colonies. This ploidy-dependent colony morphology phenotype is a result of the interaction between the defect in mother-daughter cell separation and the difference in bud site selection between haploids (axial budding pattern) and diploids (bipolar-pattern) (Voth *et al.* 2005).

We inspected the genomic sequence of the *ACE2* gene in the haploid derivative JAY291 (Argueso *et al.* 2009), and compared it to the sequence in the S288c reference genome. Only one difference was identified: The wild type *ACE2* allele in S288c contains a homopolymer run of 8 adenine nucleotides, while the mutant allele in JAY291 has 7 adenines in this region, resulting in a -1 frameshift mutation and a stop codon shortly downstream. Hence, we named the mutant allele *ace2-A7*. We then conducted reciprocal complementation tests to formally demonstrate that *ace2-A7* was the causal mutation responsible for the rough colony phenotype. The mutant alleles in haploids JAY291 and JAY292 were replaced with the wild type allele, resulting respectively in the isogenic *ACE2* strains JAY1051 and JAY1039. When these allele replacement strains were crossed to *ace2-A7* strains (Figure 2C), the resulting diploids displayed the smooth colony phenotype. This result confirmed that the wild type *ACE2* allele fully complemented the *ace2-A7* mutation and restored normal colony morphology in heterozygous diploids in this genetic background.

### LOH is the primary mechanism leading to the appearance of spontaneous rough colony isolates

After identifying the causal relationship between a mutation at the *ACE2* locus and the rough colony phenotype, we determined its DNA sequence in the five spontaneous rough colony derivatives isolated earlier in the study. We PCR-amplified and Sanger-sequenced the region containing the adenine homopolymer run in *ACE2* from JAY270, from the haploid derivatives JAY290 and JAY291, and from the rough colony isolates (Figure 2D-E). This analysis confirmed the presence of a run of 8 adenines in *ACE2* (JAY290) and 7 adenines in *ace2-A7* (JAY291). The chromatogram in the JAY270 heterozygous diploid was consistent with a mixture of *ACE2* and *ace2-A7* DNA templates being present in the sequencing reaction: single nucleotide peaks were observed at positions primer-proximal to the homopolymer run, and out-of-register double peaks were seen downstream of the seventh adenine nucleotide. The chromatograms for JAY663 (Figure 2E) and all four additional rough colony isolates (data not shown) were consistent with the presence of the *ace2-A7* frameshift mutation and absence of the *ACE2* allele. The loss of the *ACE2* allele in the diploid rough colony isolates may be explained by either copy-neutral LOH mechanisms, such as inter-homolog mitotic recombination and *de novo* -1 contraction in the adenine homopolymer run of the *ACE2* allele, or by copy-negative mechanisms such as segmental deletion spanning *ACE2* or Chr12 monosomy. To distinguish between these scenarios, we conducted quantitative digital droplet PCR analysis to determine the copy number status of the *ACE2* locus relative to genes immediately to the left (*DNM1*) and right (*YLR001C*) of the Chr12 centromere (*CEN12-L* and *CEN12-R* ddPCR probes, respectively), and relative to the *ENB1* gene, which is known to be present as single hemizygous copy in JAY270’s Chr15 (Argueso *et al.* 2009). This analysis showed that all five rough colony isolates contained two copies of *ACE2* locus DNA material (Figure 2F), thus ruling out the segmental deletion and monosomy mechanisms. 

**Figure 3:**
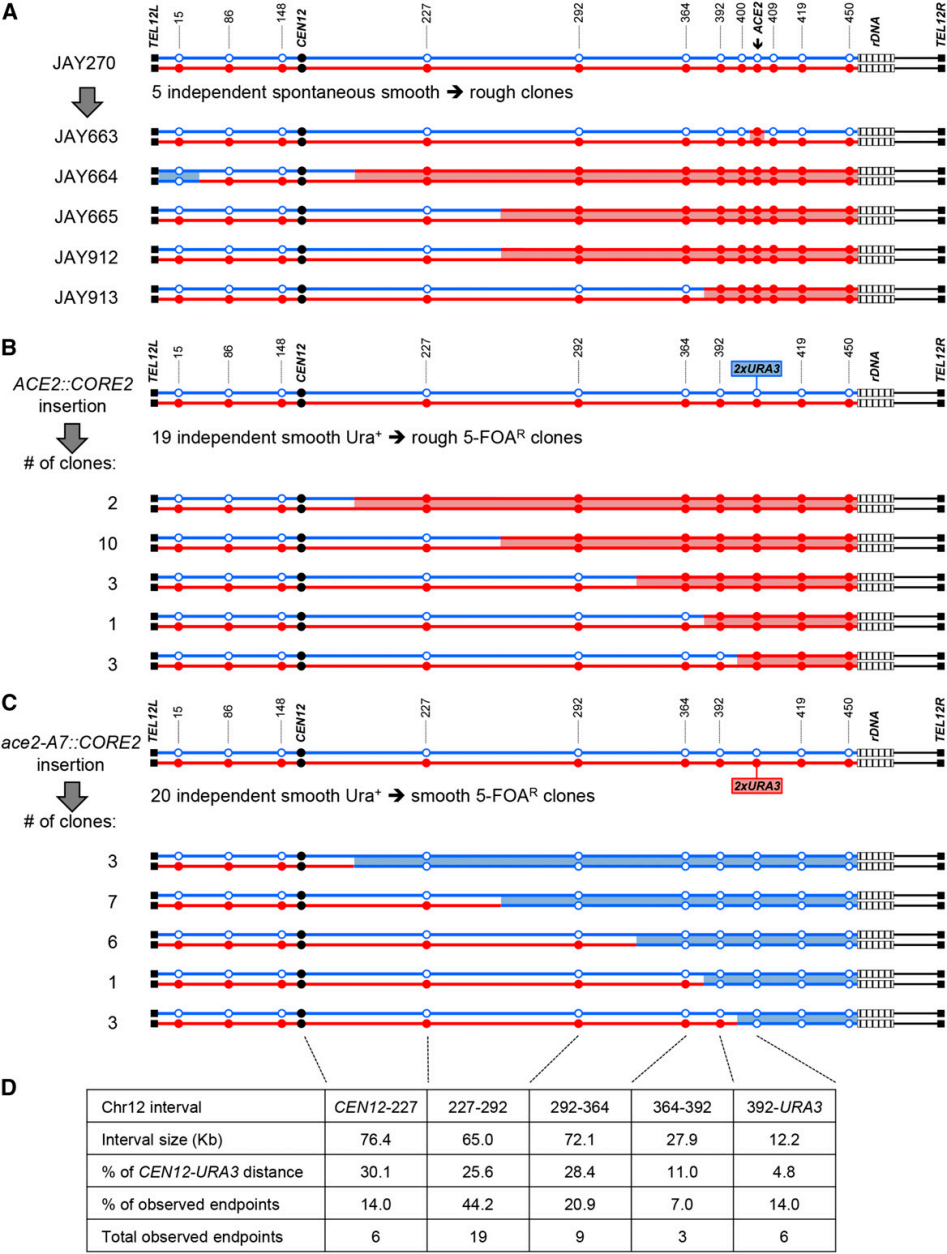
LOH tract maps of the *ACE2* and Chr12 heterozygous regions. A The genotypes at twelve phased JAY270 Chr12 HetSNP marker loci were determined using PCR and RFLP or Sanger sequencing analyses (Table S3). The approximate coordinates of the markers are shown in Kb. The Chr12 homolog containing the *ace2-A7* allele was arbitrarily designated as maternal (Chr12-M, red) and the homolog containing the wild type *ACE2* allele as paternal (Chr12-P, blue). JAY270 was heterozygous at all markers, and all rough colony isolates were homozygous for the *ace2-A7* allele. White boxes distal to the 450 Kb HetSNP represent ∼1,500 Kb of ribosomal DNA repeats (rDNA). Chr12 right arm regions distal to the rDNA do not contain any heterozygous markers in JAY270. The red or blue shading between homologs corresponds to the directions (M or P, respectively) and approximate endpoint positions of the LOH tracts (midpoint between HetSNP markers). Panels B and C show the patterns of LOH found in independent 5-FOA resistant clones derived from JAY270 with hemizygous insertions of the CORE2 cassette (*_Kl_URA3-_Sc_URA3-KanMX4*) either adjacent to the *ACE2* allele in the paternal Chr12 homolog (B) or adjacent to the *ace2-A7* allele in the maternal Chr12 homolog (C). The clones selected in B were *ace2-A7/ace2-A7* and formed rough colonies, whereas the clones selected in C were *ACE2/ACE2* and formed smooth colonies. The genotypes at the nine HetSNP loci at the indicated positions were determined by PCR-RFLP. The clones showing continuous LOH tracts were grouped according to the endpoint interval between HetSNPs, and the number of clones in each group is indicated to the left. Panel D shows a representation of the endpoint distribution among clones with Chr12 LOH. These clones were arranged in groups according to the PCR-RFLP HetSNPs marker positions used in A, B and C. The table shows the analysis of absolute interval size, relative size compared to the *CEN12* to CORE2 distance, and the number and frequency of endpoints (combined from A, B and C) for each Chr12 interval. Note that only clones with uninterrupted tracts are shown in B-C. Two clones with complex tracts (non-contiguous or bidirectional) were omitted from this figure and the endpoint distribution analysis.

LOH due to interhomolog recombination is typically a regional mutational mechanism. Interstitial tracts of homozygosity can span tens of kilobases, and terminal tracts are even longer, extending all the way to the telomeres (St Charles and Petes 2013). Therefore, in addition to being homozygous for *ace2-A7*, the rough colony isolates, if derived from interhomolog recombination, might also be homozygous for flanking HetSNPs. In contrast, copy neutral LOH caused by a *de novo* -1 contraction should be restricted to the adenine homopolymer run. We distinguished these mechanisms by determining the genotypes at eleven Chr12 HetSNPs flanking the *ACE2* locus using PCR followed by Sanger sequencing or restriction fragment length polymorphism analysis (RFLP; Table S3). The results of this analysis were compiled to produce the LOH tract maps shown in Figure 3A. As expected, JAY270 was heterozygous for all eleven markers tested. Notably, Chr12 in this strain is only heterozygous for positions to the left of the rDNA cluster (Fig. S1). This pattern is similar to that described previously for other heterozygous diploid *S. cerevisiae* genomes and is suggestive of ancestral LOH events mediated by rDNA instability (Magwene *et al.* 2011).

Analysis of the JAY663 isolate showed that, while it was homozygous for the *ace2-A7* mutation, it remained heterozygous at all other flanking markers, including those immediately proximal and immediately distal to the *ACE2* locus. Therefore, in this particular case, we were unable to distinguish between a short gene conversion tract limited to the 8.7 Kb region between the immediate flanking HetSNPs *vs.* a *de novo* -1 contraction. Despite this limitation, we favor the gene conversion mechanism, since the rate of mitotic recombination (∼10^−4^; (St Charles and Petes 2013)) is ∼10,000 fold higher than the estimated rate of single nucleotide contractions within A homopolymer runs (∼10^−8^); (Tran *et al.* 1997)).

The four remaining isolates (JAY664, JAY665, JAY912, and JAY913) were homozygous for regions well beyond the *ACE2* locus. All four LOH tracts were uninterrupted with homozygosity for nucleotides present in the Chr12 homolog that contained the *ace2-A7* allele, which we arbitrarily designated the maternal homolog (Chr12-M; red in all figures). The centromere-proximal endpoints of the LOH tracts were roughly mapped to positions ranging from 39 Kb (JAY913) to 184 Kb (JAY664) from the *ACE2* locus. On the distal side, these isolates had an additional 45 Kb of LOH that extended to the HetSNP at position 450 Kb, located 1.4 Kb proximal to the rDNA repeats. From this point, Chr12 contains ∼1,500 Kb of rDNA repeats plus another ∼600 Kb of distal homozygous single copy sequences. Since the 450 Kb HetSNP was the most distal marker in Chr12, we could not distinguish if these LOH tracts were generated as very long interstitial gene conversion events, or if they extended to the right telomere. This analysis also revealed an unexpected unselected LOH event on the left arm of Chr12 in JAY664, but in this case, it was associated with homozygosity for the nucleotide from the paternal homolog (Chr12-P, blue in all figures).

### JAY270 does not show inherently higher rates of mitotic recombination

Taken together, the results described above showed that the majority of the isolates with altered colony morphology were homozygous not only at the *ACE2* locus, but also at surrounding regions, indicating that interhomolog mitotic recombination was readily detectable in JAY270 and that it likely had a substantial role in shaping the genome of this strain. This was consistent with the uneven genomic distribution of HetSNPs (Fig. S1), with long tracts of homozygosity, suggesting that multiple LOH events accumulated in the ancestral lineage before JAY270 was isolated (Basso *et al.* 2008).

**Figure 4:**
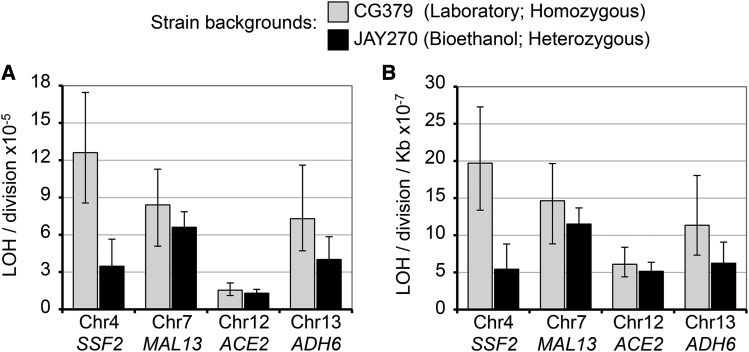
Quantitative analyses of LOH. The median absolute rates of LOH events (A) and normalized LOH rates per kilobase (respective *CEN* to CORE2 distance) (B) at four different genomic positions were measured in the CG379 laboratory strain background (gray bars) and in the JAY270 bioethanol strain background (black bars). CG379-derived diploids were homozygous at all genomic positions, except at the *MAT* locus and at the indicated marker positions. JAY270-derived diploids were heterozygous at multiple sites across the genome (Fig. S1), and at the indicated marker positions. In the X axis, Chr4, Chr7, Chr12, and Chr13 indicate diploids with hemizygous insertions of the CORE2 cassette (*_Kl_URA3-_Sc_URA3-Kan*MX4) at each of those chromosomes. The LOH rate shown for Chr12 in JAY270 was derived from the strain carrying the *ACE2*/*ace2-A7*::*CORE2* insertion (smooth Ura^+^ to smooth 5-FOA^R^ transition). Error bars indicate 95% confidence intervals (CI) for each rate measurement.

To gain a deeper understanding of the degree of genomic instability in JAY270, we conducted experiments to directly measure the rate of LOH in this strain (Figure 4A-B). Starting with a homozygous *ura3Δ/ura3Δ* derivative of JAY270 (FGY50; gift from F. Galzerani), we introduced one copy of the *_Kl_URA3-_Sc_URA3-KanMX4* CORE2 counter-selectable cassette (Zhang *et al.* 2013) at a position immediately proximal to the *ACE2* locus (1.3 Kb from the adenine homopolymer run) in either homolog. We grew cultures of strains carrying this insertion and plated the cells in media containing 5-FOA to select for clones that had lost the cassette. We performed these quantitative LOH rate measurements using the derivative carrying the hemizygous CORE2 insertion at Chr12-M, in which selected LOH produces cells with an *ACE2*/*ACE2* genotype that remain dispersed in liquid culture. The reciprocal strain, with the CORE2 insertion at Chr12-P, could not be used for this quantitative application, since the selected LOH events produces cells with the *ace2-A7*/*ace2-A7* genotype that aggregate, and thus the number of colony forming units observed in 5-FOA plates may not precisely correspond to the number of LOH-carrying cells in the population.

The median frequency of selected homozygosity for the *ACE2* allele was 1.2 × 10^−4^, and the corresponding LOH rate was calculated to be 1.5 × 10^−5^/cell division (Figure 4A). These measurements were in agreement with the unselected frequency of *ace2-A7* homozygosity estimated earlier in the study (5 rough isolates from ∼50,000 colonies). We also used hemizygous CORE2 insertions to measure LOH rates at three other positions in the genome (Chr4 near *SSF2*, Chr7 near *MAL13*, and Chr13 near *ADH6*). In order to provide a reference for comparison of LOH rates measured in JAY270, we created these same four CORE2 insertions in a conventional homozygous diploid laboratory strain background routinely used to study genomic instability mechanisms, including LOH (strain CG379; Figure 4A-B) (Morrison *et al.* 1991; Rosen *et al.* 2013; Cornelio *et al.* 2017). The measured LOH rates (Figure 4A) were either not significantly different between the two strain backgrounds at Chr12 (*P* = 0.225) and at Chr7 (*P* = 0.307), or when different, they were somewhat lower in JAY270 at Chr13 (*P* = 0.033) and Chr4 (*P* < 0.001). Overall, these direct quantitative comparisons indicated that the two strain backgrounds have similar LOH rates, and therefore their respective inherent levels of genomic instability appear to be quite comparable (*i.e.*, JAY270 is as stable as a conventional laboratory strain).

In addition, we asked whether LOH events occur more frequently at the Chr12 interval that spans *ACE2* relative to other regions of the genome. Given that most LOH tracts were suggestive of crossover or break-induced replication (BIR) recombination mechanisms (see below), we calculated the normalized LOH rate per Kb for each interval, by dividing the absolute LOH rate by the distance between the centromeres and the respective CORE2 insertion sites. This analysis (Figure 4B) indicated that all four intervals have relatively similar rates of LOH, and that recombination near the *ACE2* locus does not appear to be elevated by the presence of the rDNA repeats on its distal side.

We also used the 5-FOA^R^ selection approach to characterize the qualitative nature of Chr12 LOH tracts in a larger set of independently selected clones. In contrast to the quantitative rate assays above, the difference in cell aggregation phenotype between LOH leading to *ACE2/ACE2* *vs.* *ace2-A7/ace2-A7* genotypes was not a concern. Thus, we used nine PCR-RFLP markers to map LOH tracts from 5-FOA^R^ clones derived from both CORE2 insertions, at Chr12-P (Figure 3B) or at Chr12-M (Figure 3C). The LOH tracts selected from both strains resembled each other’s, and the tracts observed in the five initial spontaneous rough colony isolates (Figure 3A). All 41 selected Chr12 LOH clones were heterozygous for the left arm, and 39 of them had uninterrupted LOH tracts going from M/P to P/P or M/M on the right arm, starting at positions between *CEN12* and *ACE2*, and extending up to the 450 Kb HetSNP. The two remaining clones had more complex tracts, including interruptions or homozygosity for the homolog opposite to the direction of selection (data not shown). The predominant LOH tract patterns were consistent with interhomolog mitotic crossover or BIR mechanisms.

Finally, we also investigated the distribution of LOH endpoints on Chr12. The region between *CEN12* and *ACE2* was divided in five intervals delimited by PCR-RFLP HetSNP markers. The distribution of endpoints found at these intervals was not significantly different between the strains carrying the CORE2 insertion at Chr12-P and Chr12-M (χ^2^ = 0.855; *P* = 0.93), suggesting that both homologs shared similar mitotic recombination properties. This similarity allowed us to pool the endpoint distribution data from the 19 5-FOA^R^ clones derived from the Chr12-P insertion to the endpoints from 20 5-FOA^R^ clones derived from the Chr12-M, and four of the original spontaneous rough clones (JAY664, JAY665, JAY912, JAY913). We compared the total number of endpoints leading to LOH observed within each interval to the expected distribution if endpoints were allocated purely as a function of the sizes of the physical intervals. This analysis indicated that the observed endpoint distribution was significantly different from this simple model (χ^2^ = 18.51; *P* = 0.001) (Figure 3D), suggesting the possible presence of a mild hotspot for initiation of mitotic recombination, particularly between the 227 and 292 Kb HetSNP markers.

## Discussion

### Rough colony phenotype and mutation in *ACE2*

We showed that a frequent colony morphology transition in JAY270 resulted from mitotic recombination events at a region of Chr12 heterozygous for a mutation in the *ACE2* gene. Importantly, *ace2-A7/ace2-A7* homozygosity also significantly accelerated the sedimentation of yeast cells in liquid culture, an undesirable trait that is typically associated with decreased fermentation kinetics and clogging of centrifuges and pipes at distilleries that use yeast recycling in fed-batch sugarcane bioethanol production (Paulino de Souza *et al.* 2018; Della-Bianca *et al.* 2013).

The *ACE2* gene encodes a transcription factor that controls the expression of at least 20 genes involved in septum destruction and mother-daughter cell separation (Weiss 2012; Doolin *et al.* 2001). The rough colony phenotype was easily discernible in *ace2-A7/ace2-A7* diploids, but was not observed in *ace2-A7* haploids. This ploidy-specificity of the colony morphology phenotype caused by loss of *ACE2* function has been previously attributed to the difference in budding patterns between diploid and haploid cells (Voth *et al.* 2005). Diploid cells typically display a bipolar pattern of budding, which when combined with cell-separation defects result in textured colony morphology. In contrast, haploid cells follow an axial budding program, during which each new bud emerges in a position adjacent to the previous bud scar. Mutation of the *BUD4* gene gives rise to haploids that display a bipolar budding pattern typical of diploid cells. Voth *et al.* 2005 showed that *ace2bud4* double mutant haploids display bipolar budding and a rough colony appearance indistinguishable from *ace2/ace2* diploids. Additionally, mutations in *ACE2* regulators, such as *CBK1*, *HYM1*, *KIC1*, *MOB2*, cause similar phenotypes including defects in cell separation and altered colony morphology.

The *ace2-A7* −1 frameshift allele found in JAY270 leads to a premature stop codon resulting in a truncated, likely inactive, protein that lacks three zinc finger domains and a nuclear localization sequence (McBride *et al.* 1999). Another heterozygous diploid industrial strain, FostersB used in brewing (Borneman *et al.* 2011), has a +1 frameshift variant in the same adenine homopolymer region of *ACE2* (*ace2-A9* allele), suggesting that it is also vulnerable to phenotypic changes caused by LOH spanning this locus.

Interestingly, *de novo* mutations in *ACE2* have been previously identified in multiple bioreactor and chemostat experimental evolution studies using *S. cerevisiae* haploids (Oud *et al.* 2013; Koschwanez *et al.* 2013; Ratcliff *et al.* 2015; Hope *et al.* 2017). The *ace2-A7*, *ace2-A9*, and additional loss-of-function alleles were identified in aggregating, fast-sedimenting adapted clones. This acquired multi-cellularity trait is thought to have provided them with enhanced fitness under the specific laboratory conditions where they evolved. In the specific case of JAY270 in the bioethanol distillery environment, it is not known if or how the switch between dispersed to aggregated cell growth states has an effect on fitness. However, it is possible that JAY270’s heterozygous *ACE2/ace-A7* genotype may offer it the benefit of versatility, by giving individuals in the population the ability to quickly access the aggregated state through LOH. This may provide a short-term adaptive solution as cells encounter various environmental challenges during industrial fermentation (Basso *et al.* 2008). Individuals in the population could then later return to the dispersed state through expansion or contraction of the adenine run (Tran *et al.* 1997), or through sporulation and mating to an *ACE2* haploid. Interestingly, a recent microsatellite-based phylogenetic study of yeast strains isolated from sugarcane bioethanol distilleries reported that within a cluster of isolates that were genetically similar to strain PE2 (the parent of JAY270), some of the isolates formed rough colonies while others had smooth colonies (Reis *et al.* 2017). This observation suggests the possibility that smooth-to-rough and perhaps also rough-back-to-smooth, transitions could actually be occurring within PE2 populations in distillery environments.

### Analyses of spontaneous and selected LOH clones

Mapping of Chr12 heterozygosity in 44 spontaneous and selected clones revealed long tracts of LOH extending for several Kb and spanning hundreds of HetSNPs in addition to the *ACE2* locus (Figure 3). Overall, the pattern of recombination observed was simple and consistent with the majority of events being the result of allelic mitotic crossovers or BIR (Symington *et al.* 2014; Kramara *et al.* 2018). However, we disfavor the BIR mechanism in this case, because the efficiency of BIR is greatly reduced (<40%) when the segment of DNA to be synthesized is longer than ∼100 Kb (Donnianni and Symington 2013). In the clones characterized here, BIR would require DNA synthesis across extremely long Chr12 DNA segments, including regions proximal to the 450 Kb HetSNP, across ∼1,500 Kb of the highly repetitive rDNA region, and additional ∼600 Kb to reach the right telomere. Thus, completing BIR through this specific region would be particularly challenging, while resolving the recombination intermediates toward the crossover outcome would not.

The *ACE2* gene is positioned fairly close (∼50 Kb proximal) to the most unstable region of the yeast genome, the rDNA repeats (Ganley and Kobayashi 2014). We asked whether this relative proximity to the rDNA repeats at Chr12 could be leading to an elevated incidence of recombination involving the *ACE2* locus. The normalized rate of mitotic recombination measured at *ACE2* was very similar to or lower than the rates measured at other regions of the genome (Figure 4B), thus we conclude that the rDNA does not destabilize the centromere-proximal regions of the right arm of Chr12. This result was consistent with previous studies that showed a ∼10 fold higher rate of recombination on the distal side of the rDNA compared to the proximal side (Casper *et al.* 2008), and with Chr12 distal tracts of LOH detected in several wild *S. cerevisiae* isolates (Magwene *et al.* 2011).

Surprisingly, our analysis uncovered one rough clone (JAY664) that carried two coincident LOH events in each arm of Chr12 and in opposite directions (the selected [*ace2-A7/ace2-A7*] M/M LOH in the right arm, and an unselected P/P LOH in the left arm). The rate of LOH that we measured at the right arm of Chr12 was approximately 1.5 × 10^−5^, thus the rate for two independent events on this chromosome is predicted to be extremely rare (∼10^−10^). Intriguing observations of elevated rates of coincident mitotic recombination in yeast have been reported previously and generally interpreted as linked to a subpopulation of recombination-prone cells (Fogel and Hurst 1963; Minet *et al.* 1980; Montelone *et al.* 1981; Golin and Esposito 1984; Golin and Tampe 1988; Freeman and Hoffmann 2007; Forche *et al.* 2009). We speculate that JAY664 may have been derived from an analogous genome-wide instability mechanism. Further work offering supporting evidence in this regard is in progress and will be described elsewhere.

### Genomic instability in JAY270 relative to a conventional laboratory strain background

Finally, the rates of LOH measured at all four genomic regions in JAY270 were comparable to the rates at the same positions in the CG379 laboratory strain background (Figure 4). The rates of LOH observed in this study in both strains were also compatible with the rates of allelic mitotic recombination reported in the literature (St Charles and Petes 2013). This result is significant because industrial yeast strains, including PE2, generally have a reputation for having inherently unstable genomes (de Amorim *et al.* 2017). The example characterized in our study suggests that, while karyotype instability is indeed frequently detected in yeast clones isolated from different industrial settings, the genomes of this group of strains may not necessarily be inherently less stable than those of conventional laboratory strains. This misconception may stem from the structural complexity of the genomes of wild and industrial isolates that make recombination between pairs of polymorphic homologs detectable through a simple karyotype analysis. These same recombination events, however, produce no net genetic change when occurring between perfectly identical pairs of homologs in homozygous laboratory diploids. Since allelic interhomolog mitotic recombination is not detectable in most conventional laboratory experimental settings, these strains may appear to be relatively stable, when in reality, recombination is also happening in them at similar rates. We believe that, at least in the case of JAY270, the high genomic instability reputation is not deserved, and the same might also be true for other industrial strains.
